# Infected Pseudoaneurysm Associated With Umbilical Depression at the Puncture Site Due to Perclose ProStyle Suture®: Restoration for Infection Prevention

**DOI:** 10.7759/cureus.80621

**Published:** 2025-03-15

**Authors:** Daisuke Yamazaki, Mitsunori Yuzurihara

**Affiliations:** 1 Cardiology, Akita Cerebrospinal and Cardiovascular Center, Akita, JPN

**Keywords:** complication, endvascular treatment, infected femoral artery pseudoaneurysm, perclose vascular device, thin patients

## Abstract

Percutaneous coronary intervention is now commonly performed using the transradial artery approach, but endovascular therapy (EVT) is still often performed via the femoral artery (FA). Vascular closure devices have been developed and are commonly used to achieve hemostasis at the FA puncture site. However, there have been some reports of infection complications associated with the use of vascular closure devices. When Perclose ProStyle Suture® (Abbott, Abbott Park, IL, USA) is used for hemostasis in thin patients, the puncture site may become umbilical depressed. We report three cases of umbilical puncture site depression with the Perclose ProStyle Suture®.

Case 1 was a 78-year-old male with a body mass index (BMI) of 18 kg/m^2^ and a thin build who underwent EVT for peripheral arterial disease with rest pain. Stents were implanted in the stenosis of both external iliac arteries and the occluded lesion of the right superficial femoral artery (SFA) via the left FA approach. The puncture site was sutured with Perclose ProStyle Suture®, but the puncture site became depressed like a navel. One month later, he visited the hospital with pus discharge from the left FA puncture site, and one week later, he visited the hospital with bleeding from an infected pseudoaneurysm. Although vascular repair surgery was performed, it recurred a month later, and thus the infected tissue was debrided and vascularization was performed using a bovine pericardial patch.

Case 2 was an 88-year-old female with a BMI of 18 kg/m^2^ who was thin and had intermittent claudication. EVT was performed on the occluded lesion in the left SFA. A stent was implanted in the left SFA via the right FA, and the puncture site was hemostatized with Perclose ProStyle Suture®. Two weeks later, she was referred to our institution because of bleeding from the puncture site in her right FA, and a contrast computed tomography revealed an infected pseudoaneurysm. She was referred to vascular surgery, where the infected area was debrided and vascular repair surgery was performed.

Case 3 was a 72-year-old male (BMI 18.4 kg/m^2^) with intermittent claudication. EVT was performed for a severe stenosis lesion in the right SFA. A stent was implanted in the right SFA via the left FA approach, and the puncture site was hemostatized using Perclose ProStyle Suture®. The puncture site then became depressed like a navel. Based on the experience of cases 1 and 2, the subcutaneous tissue around the suture was incised, the suture was buried in the subcutaneous tissue, and the dermis was sutured with 5-0 absorbable sutures. The patient is being followed up as an outpatient, and there is no evidence of infection, and the condition is good.

Due to the design of the device, the Perclose ProStyle Suture® is designed to suture approximately 1 cm proximal to the vessel wall. Therefore, in such cases, where the patient is thin and has thin subcutaneous tissue, the suture is placed close to the dermis, and when ligated, the dermis is depressed like a navel. When the suture is close to the dermis, the risk of vascular infection increases; therefore, it is preferable to perform the repair so that the suture is buried in the subcutaneous tissue.

## Introduction

Percutaneous coronary intervention (PCI) is commonly performed using the radial artery approach, but the femoral artery (FA) approach is still often chosen when treating complex lesions (heavily calcified, chronic total occlusions, etc.) or performing endovascular therapy (EVT). Vascular closure devices have been developed and are commonly used to reduce bleeding complications and shorten the time to postoperative rest after FA puncture. Although hemostasis has become easier after FA access, there have been reports of complications such as infection with the use of vascular closure devices [1,2]. The infection complication rate with the use of vascular closure devices has been reported to be 0.6% [3]. In cases of an infected pseudoaneurysm, conservative treatment often does not cure the condition, and in most cases, surgical treatment is required [4].

After performing PCI or EVT via the FA approach, we use Perclose ProStyle Suture® (Abbott, Abbott Park, IL, USA) to achieve hemostasis at the puncture site. However, in rare cases, the knot of the suture thread forms in the depth of the epidermis, causing the puncture site to sink inward like a navel. We report two cases in which the skin at the puncture site became navel-shaped after hemostasis with Perclose ProStyle Suture®, resulting in the formation of an infected pseudoaneurysm, and one case in which measures were taken to prevent this.

## Case presentation

Case 1

A 78-year-old man with Parkinson's disease (height 163 cm, weight 49 kg, body mass index [BMI] 18 kg/m^2^) presented with a chief complaint of rest pain in both lower limbs. A computed tomography (CT) scan of the lower limbs revealed bilateral external iliac artery stenosis and bilateral superficial femoral artery (SFA) occlusion. Because the underlying disease made exercise therapy difficult, EVT was chosen. The left FA was punctured, and a Parent Plus 60Ⓡ (Medikit, Tokyo, Japan) with an external diameter of 2.8 mm and an internal diameter of 6 Fr was inserted. Two S.M.A.R.T. CONTROLⓇ (Cordis, Santa Clara, CA, USA) were implanted in the stenotic lesions of the bilateral external iliac arteries, and ELUVIAⓇ (Boston Scientific, Marlborough, MA, USA) was implanted in the occluded lesion of the right SFA. The puncture site was closed with Perclose ProStyle Suture®, and although hemostasis was achieved, the knot of the suture was slightly visible above the skin, causing a depression in the skin. We do not have a photograph of this, but it looked like case 3. In the second EVT, a stent was implanted in the occluded area of the left SFA through a right FA approach.

**Figure 1:**
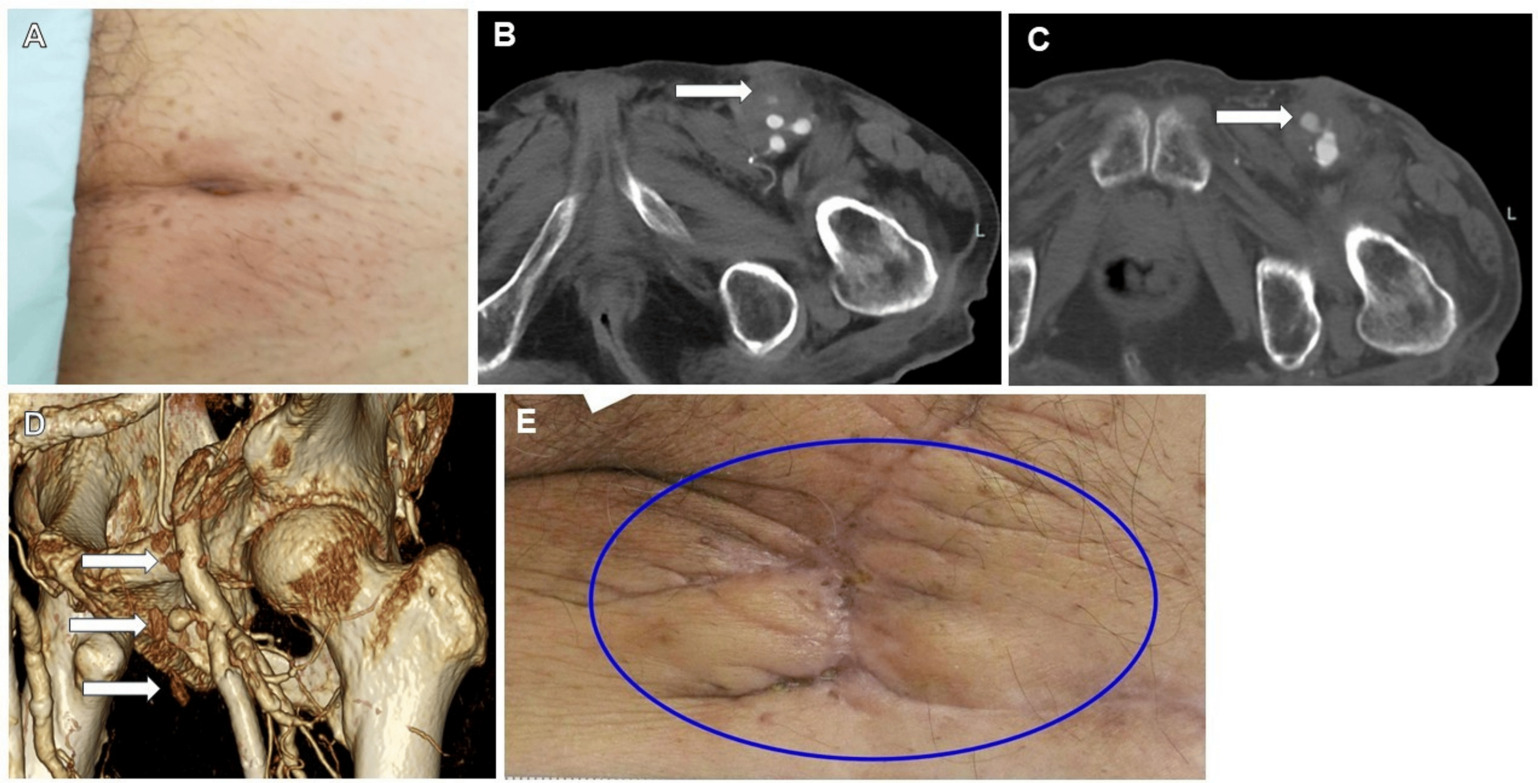
Puncture site photographs and CT images of case 1. A: Photograph of the puncture site at the left femoral artery (FA) after removal of the suture. There was only mild redness and swelling, and blood tests showed no signs of inflammation. B: Contrast CT scan taken when the patient presented to the hospital with bleeding from the puncture site. A pseudoaneurysm (arrow) was found at the anterior aspect of the FA, and a soft tissue shadow with low contrast was observed around it. C: CT image of the second infected pseudoaneurysm. A larger aneurysm than the first was observed (arrow), and a low-contrast soft tissue shadow was seen around it. D: 3D CT image of the second infected pseudoaneurysm. An oozing hemorrhage was also seen around the pseudoaneurysm (arrows), suggesting an extension of the area of infected tissue. E: Photograph of the wound after the second vascular repair and skin flap surgery. There is scarring but no evidence of infection (circle). CT, computed tomography

One month later, the patient presented to the hospital with redness and pus around the left FA puncture site and a small amount of suture protruding from the skin. On plain CT, the inflammatory findings were localized around the epidermis, and there was no elevation of white blood cells or C-reactive protein in the blood test, and thus the suture was removed, antibiotics were prescribed, and the patient was observed for recovery. Figure 1A shows the puncture site after suture removal. He was examined one week later due to bleeding from the puncture site in the left FA. The blood test showed an inflammatory response with white blood cells of 8750/μL, neutrophils of 76.3%, and C-reactive protein of 4.0 mg/dL. Contrast CT revealed an infected pseudoaneurysm at the puncture site (Figure 1B), and the patient was referred to a vascular surgeon at a nearby hospital. On the same day, he underwent drainage and vascular repair surgery for the infected pseudoaneurysm. A pseudoaneurysm and nasopharyngeal culture showed methicillin-resistant Staphylococcus aureus. However, one month later, the infected pseudoaneurysm recurred, and he was referred back to the vascular surgery department. The contrast CT at that time showed recurrence of the infected pseudoaneurysm and inflammatory findings in the surrounding area (Figure 1C). Figure 1D shows the 3D CT, which also showed hemorrhage around the pseudoaneurysm, suggesting that the area of infected tissue had expanded. The infected tissue was extendedly debrided, and a bovine pericardial patch was used to form an infected vessel. After vacuum-assisted closure therapy for one month, the wound was closed, and the infected pseudoaneurysm did not recur. The final appearance of the wound is shown in Figure 1E.

Case 2 

**Figure 2:**
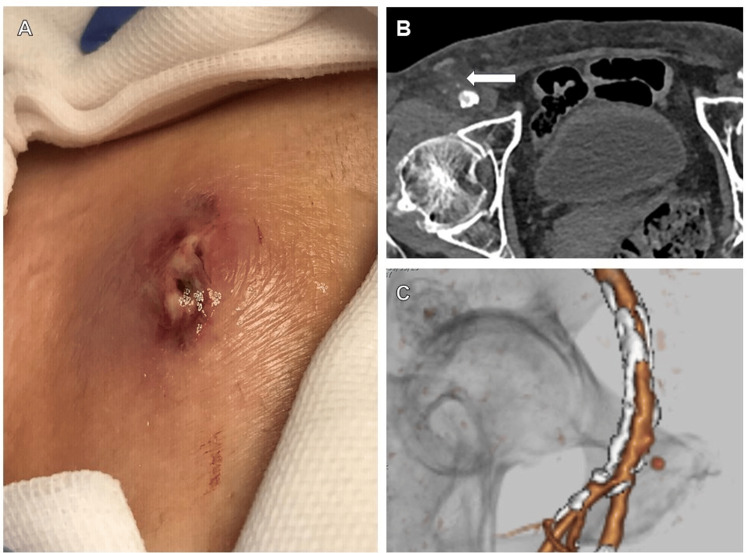
Photographs and CT images of the puncture site in case 2. A: Photograph of the puncture site in the right femoral artery. Redness, swelling, and pus are present. B: Contrast-enhanced CT image of the puncture site. A soft tissue shadow with mild contrast enhancement can be seen around the puncture site. C: Contrast-enhanced 3D CT image. Although it was small, with a diameter of 3 mm, a pseudoaneurysm is shown in front of the puncture site. CT, computed tomography

An 88-year-old female (height 143 cm, weight 37 kg, BMI 18 kg/m^2^) with underlying hypertension and dyslipidemia was referred to our hospital with intermittent claudication as the main complaint. A lower limb CT angiography revealed left SFA occlusion. Because she was elderly and exercise therapy was difficult, we decided to perform EVT. The right FA was punctured, a guiding sheath (Parent Plus 60Ⓡ) was inserted, and an ELUVIAⓇ stent was implanted in the occluded left SFA. The puncture site was hemostatized using Perclose ProStyle Suture®. However, as in case 1, the knot slightly protruded from the epidermis, and the puncture site was indented like a navel (in case 2, the area where the epidermis was indented during hemostasis was not photographed). When the knot was pushed in, it hid deeper than the dermis, and thus we decided to observe the puncture site. Two weeks after EVT, the patient presented to the hospital with bleeding from the puncture site. The blood test showed an inflammatory response with white blood cells of 9,000/μL, neutrophils of 88.6%, and C-reactive protein of 4.6 mg/dL. Although the puncture site had stopped bleeding, it was swelling with pus (Figure 2A), and contrast CT showed a pseudoaneurysm at the puncture site with evidence of inflammation around it (Figures 2B, 2C). She was referred to a vascular surgeon at a nearby hospital for the diagnosis of an infected pseudoaneurysm and underwent surgical debridement of the infected area and vascular repair. Methicillin-susceptible Staphylococcus aureus was detected in the blood culture. The wound was left open, and vacuum-assisted closure therapy was continued for one month, after which the wound was closed with a skin flap. There was no recurrence of infection, and the patient's condition improved.

Case 3

**Figure 3:**
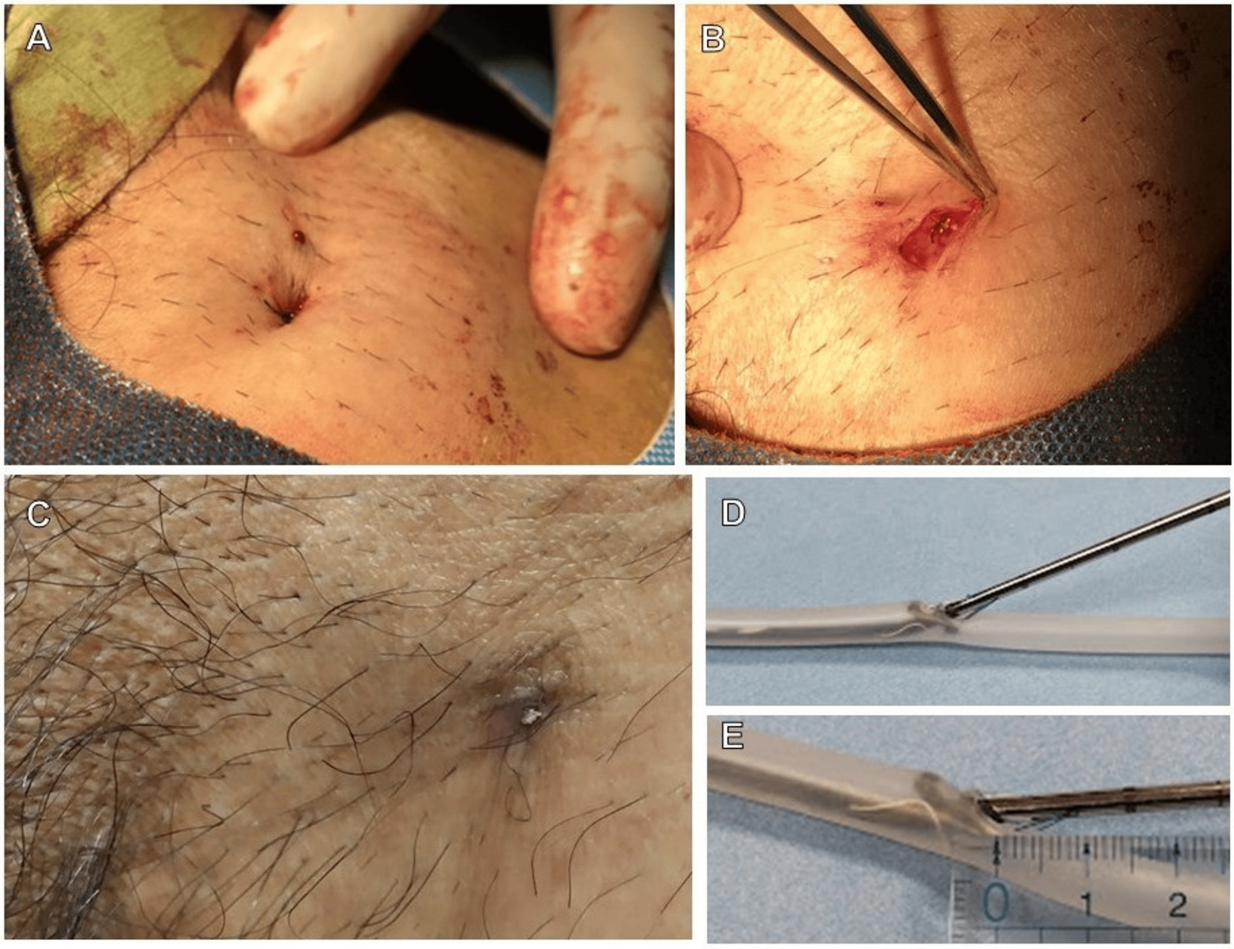
The puncture site and follow-up after hemostasis of case 3. The hemostasis process using the Perclose ProStyle Suture®. A: After hemostasis of the puncture site in the left femoral artery. The suture is exposed on the body surface, and the skin is depressed in the shape of a navel. B: During repair. An approximately 1-cm incision was made around the puncture site, and the suture and surrounding tissue were separated. The epidermis was sutured with 5-0 absorbable sutures in a buried suture so that the sutures of the Perclose Prostyle Suture® remain intact and do not come in contact with the outside. C: Appearance of the puncture site during outpatient follow-up. The suture has partially formed a scab, but there are no signs of infection (erythema and swelling, etc.). D: Appearance of the suturing a blood vessel model with Perclose ProStyle Suture®. Two needles protrude from the proximal side of the Perclose Prostyle Suture® and suture the area around the puncture site. E: The needles protrude approximately 1 cm proximal to the blood vessel wall, and thus the knot of the suture is approximately 1 cm proximal to the blood vessel wall.

A 72-year-old male (height 169 cm, weight 52.9 kg, BMI 18.4 kg/m^2^) with hypertension and lumbar spinal canal stenosis was referred to us after an ankle brachial index of 0.71 was measured on the right side during a blood pressure pulse wave test due to intermittent claudication. Lower limb contrast CT showed stenosis of the right SFA. Because the patient was unable to perform full exercise therapy due to the lumbar spinal canal stenosis, EVT was planned. The left FA was punctured and a Parent Plus 60Ⓡ was inserted, and an ELUVIAⓇ stent was placed in the stenotic lesion of the right SFA. The puncture site in the left FA was hemostatized using Perclose ProStyle Suture®, but, as in cases 1 and 2, the knot of the suture thread formed at the level of the epidermis, and the puncture site was depressed like a navel (Figure 3A). Because we experienced complications of infectious pseudoaneurysms in cases 1 and 2, we repaired the exposed knot and the umbilical skin depression. After additional local anesthesia, we made an approximately 1-cm incision around the puncture site and dissected the knot and surrounding subcutaneous tissue that had become involved (Figure 3B). After separation, the knot remained within the subcutaneous tissue layer, and the depression of the epidermis at the puncture site was repaired. The epidermis was sutured with 5-0 absorbable sutures in a buried suture so that the sutures would not communicate with the outside. The patient was then observed in the outpatient clinic for any changes at the puncture site. Even after three months of the EVT, although some areas of the wound showed signs of crusting, there was no evidence of infection (erythema, swelling, etc.), and the patient was in good condition (Figure 3C).

## Discussion

The FA approach is the most basic approach when performing PCI or EVT. In the past, manual compression was used to achieve hemostasis, and long periods of bed rest were required after hemostasis. The sheath inserted into the FA during treatment was usually 6-7 French, and bleeding complications were sometimes observed. To reduce the need for prolonged bed rest and bleeding complications, various hemostatic devices have been developed by various companies, including Perclose ProGlide® (Abbott, Abbott Park, IL, USA), Angio-Seal® (St. Jude Medical, St. Paul, MN, USA), and Exoseal® (Cordis Corporation, Milpitas, CA, USA) [5]. While it has become easier to achieve hemostasis at the FA puncture site, there have also been reports of infection complications due to vascular closure devices [6-8], but no detailed discussion of the causes of infection has been confirmed.

From case reports, it is clear that infected pseudoaneurysms are almost never cured with conservative treatment such as antibiotics and always require surgical treatment [6-8]. There is a case report in which catheter treatment was used to treat infectious pseudoaneurysms in exceptional cases due to the patient's ability to tolerate surgery, but these are extremely rare cases [9]. If vascular repair surgery is performed due to infectious complications, the benefits of minimally invasive catheter treatment are lost, and the length of hospital stay is prolonged. Thus, infected pseudoaneurysms are a complication that should be avoided.

We experienced two cases of infected pseudoaneurysms that developed after the puncture site was sutured, with the epidermis being depressed like a navel. Therefore, we thought that the depressed puncture site might cause the distance between the outer surface and the FA to shorten, leading to infection. Having experienced these two cases, in the third case, we prevented the development of an infected pseudoaneurysm by making an incision around the puncture site, pushing the suture thread into the subcutaneous tissue layer, and repairing the depressed skin. When considering the cause of the skin indentation, the BMI of all three patients was 18 kg/m2, indicating that the patient was underweight, and the subcutaneous tissue between the skin surface and the FA at the puncture site was thin. Figure 3D shows a model of how sutures are connected using Perclose ProStyle Suture®. The Perclose ProStyle Suture® has two needles that pierce the area around the puncture site and stitch it together. The needle is sewn at the base of the needle, and according to Figure 3E, the suture is about 1 cm proximal to the blood vessel wall, so the subcutaneous tissue in front of the blood vessel wall is also sewn. Therefore, in cases such as the three cases we reported, where the patient is thin and has thin subcutaneous tissue, the suture knot will be close to the epidermis, and by suturing the tissue just under the epidermis, it will sink inward like a navel. The suture is continuous with the vessel wall, and when the skin is depressed, the distance between the vessel wall and the outside also becomes shorter. In addition, the groin is an easily infected area. Thus, if the skin is depressed and the suture knot is exposed, the risk of infection is extremely high. No other literature has discussed the mechanism of umbilical depression with the use of this Perclose ProStyle Suture® or its association with infected pseudoaneurysms. The depth to which the knot was buried at the time of repair was not clearly determined, but the depression of the umbilical fossa was repaired by incising the subcutaneous tissue around the knot, suturing the buried dermis, and cutting the suture from the outside. Even if hemostasis is sufficient in such cases, the puncture site should be repaired. This repair technique is not an evidence-based procedure but a preventive measure based on our hypothesis.

## Conclusions

In conclusion, when Perclose ProStyle Suture® is used to achieve hemostasis in thin patients, there is a risk of exposure of the suture site or depression of the puncture site. In such cases, because of the proximity of the suture to the body surface, infection can spread to the vessel wall via the suture and cause an infected pseudoaneurysm. Therefore, if the puncture site is umbilically depressed, it should be repaired so that the knot is cut off from the outer surface, and close follow-up is then necessary to ensure that there are no complications of infection.
